# Bacillus Calmette-Guérin immunotherapy induces an efficient antitumor response to control murine melanoma depending on MyD88 signaling

**DOI:** 10.3389/fimmu.2024.1380069

**Published:** 2024-05-21

**Authors:** Vinícius M. Borges, Fábio V. Marinho, Christiane V. A. Caldeira, Nina M. G. P. de Queiroz, Sergio C. Oliveira

**Affiliations:** ^1^ Departamento de Imunologia, Instituto de Ciências Biomédicas, Universidade de São Paulo, São Paulo, Brazil; ^2^ Departamento de Bioquímica e Imunologia, Instituto de Ciências Biológicas, Universidade Federal de Minas Gerais, Belo Horizonte, MG, Brazil; ^3^ Institut Pasteur de São Paulo, São Paulo, Brazil

**Keywords:** Bacillus Calmette-Guérin (BCG), cancer immunotherapy, melanoma, innate immune pathways, MyD88, TLRs, cGAS-STING

## Abstract

Bacillus Calmette-Guérin (BCG) is the first line treatment for bladder cancer and it is also proposed for melanoma immunotherapy. BCG modulates the tumor microenvironment (TME) inducing an antitumor effective response, but the immune mechanisms involved still poorly understood. The immune profile of B16-F10 murine melanoma cells was assessed by infecting these cells with BCG or stimulating them with agonists for different innate immune pathways such as TLRs, inflammasome, cGAS-STING and type I IFN. B16-F10 did not respond to any of those stimuli, except for type I IFN agonists, contrasting with bone marrow-derived macrophages (BMDMs) that showed high production of proinflammatory cytokines. Additionally, we confirmed that BCG is able to infect B16-F10, which in turn can activate macrophages and spleen cells from mice in co-culture experiments. Furthermore, we established a subcutaneous B16-F10 melanoma model for intratumoral BCG treatment and compared wild type mice to TLR2^-/-^, TLR3^-/-^, TLR4^-/-^, TLR7^-/-^, TLR3/7/9^-/-^, caspase 1^-/-^, caspase 11^-/-^, IL-1R^-/-^, cGAS^-/-^, STING^-/-^, IFNAR^-/-^, MyD88^-/-^deficient animals. These results *in vivo* demonstrate that MyD88 signaling is important for BCG immunotherapy to control melanoma in mice. Also, BCG fails to induce cytokine production in the co-culture experiments using B16-F10 and BMDMs or spleen cells derived from MyD88^-/-^ compared to wild-type (WT) animals. Immunotherapy with BCG was not able to induce the recruitment of inflammatory cells in the TME from MyD88^-/-^ mice, impairing tumor control and IFN-γ production by T cells. In conclusion, MyD88 impacts on both innate and adaptive responses to BCG leading to an efficient antitumor response against melanoma.

## 1 Introduction

Melanoma is the most aggressive type of skin cancer, with a highly metastatic potential in 3.5% of cases (1). At the earliest stages of progression, the lesion is localized, and surgery for these cases is highly effective and curative. However, remnant malignant cells in the invasive border after tumor excision or circulating metastatic cells can lead to melanoma recurrence (2). In addition, the tumor can progress to an unresectable advanced stage associated with poor prognosis and high disease lethality. Therapeutic heterogeneity is required to cope with clinical condition diversity and resistant mechanisms that impact on melanoma treatment (3), raising the interest to develop new therapies. To face this challenge, BCG (Bacillus Calmette-Guérin) immunotherapy, the gold standard adjuvant treatment for bladder cancer applied in clinics for more than 40 years (4), also stands out as an auxiliary therapy recommended for the treatment of unresectable melanoma cutaneous metastases (5). Direct injection of BCG into metastatic melanoma lesions in the skin induces the complete or partial regression of injected tumors as well as elicits a systemic effect on the distant non-injected metastases (6, 7). However, the main limitation to BCG therapy improvement is the limited knowledge about the specific mechanism of BCG-mediated tumor immunity, which remains poorly understood in bladder cancer and even less in melanoma.

The composition of the BCG cell wall is highly immunogenic (8) due to the large amount of Toll-like receptor 2 (TLR2) ligands, such as lipomannan (9), dimannoside (PIM_2_) and hexamannoside (PIM_6_) phosphatidylinositol mannosides (10), as well as muramyl dipeptide derivatives which can also activate TLR4 (11) or nucleotide-binding oligomerization domain-containing protein 2 (NOD2) (12). The cytosolic heat shock proteins HSP65 and HSP70 found in *Mycobacterium tuberculosis* which elicit a TLR4-mediated response (13) is also present in BCG. Immunogenic activity was observed in a BCG nucleic acid extracted fraction composed by 70% of DNA and 28% of RNA (14) containing CRISPR cognates, small noncoding RNAs (sncRNAs) (15), and unmethylated CpG motif-enriched bacterial DNA (16) that can function as TLR3, TLR7, and TLR9 ligands, respectively. The majority of TLRs depends on the MyD88 adaptor molecule, except for TLR3 that recruits TRIF and TLR4 that depends on both MyD88 and TRIF signaling (17). Accordingly, BCG promotes the activation of macrophages and dendritic cells in a MyD88-dependent manner (16).

BCG also produces moderate amounts of cyclic di-AMP, capable to activate Stimulator of interferon genes (STING) (18). The inflammasome (19) and cGAS-STING pathways (18) have a minor role in BCG-induced response due to RD-1 genomic region deletion with consequent loss of secretion system and reduced virulence (20). Despite BCG’s limited ability to release nucleic acids into cytosol, the dead cells present in the tumor microenvironment (TME) might be able to activate the inflammasome (21) and cGAS-STING pathways (22) in bystander cells. Interestingly, the overexpression of the inflammasome sensor absent in melanoma 2 (AIM2) in MB49 murine bladder tumors favors BCG immunotherapy (23). The activation of the inflammasome culminates in the cleavage and release of the active forms of IL-1β and IL-18, those receptors bear the Toll/Interleukin-1 receptor (TIR) domain and rely on MyD88 recruitment to signal in activated cells (17). Moreover, the treatment of murine bladder cancer with BCG intravesical instillations culminates in increased expression of STING (24). In addition, the application of a BCG overexpressing cyclic di-AMP to activate STING signaling enhances the induction of type I interferon and improve the antitumor efficacy (25). Therefore, a better understanding of the immune mechanisms involved in BCG immunotherapy is essential to improve its use on melanoma.

A previous study from our group showed that BCG acts modulating the TME in subcutaneous MB49 bladder tumor model through the recruitment of antitumor cells, such as inflammatory macrophages, neutrophils, CD8^+^ T cells and NKT cells. Taken together, the *in vitro* response to BCG, TME modulation, and tumor remission promoted by BCG in MB49 tumors are intrinsically dependent on MyD88 signaling. However, the specific MyD88-upstream receptor was not identified, whereas several TLRs and IL-1R tested did not appear to be involved in BCG-induced MB49 tumor control (26). The studies related to BCG immunotherapy in melanoma revealed an increase in the production of inflammatory cytokines (IFN-γ, TNF-α, TNF-β, IL-15, IL-1β, IL-6, and IL-32), chemokines (CCL2, CCL18, CXCL9, CXCL10, and CXCL11), as well as stress-induced molecules (BTN3A1 and MICB) after BCG intratumoral injection that correlates with regression of cutaneous melanoma metastases (27). Also, increase in different immune cell subsets such as macrophages (7), Batf3-dependent dendritic cells (cDC_1_) (7, 28), γδ T cells (27), CD8^+^ T cells (28), and natural killer (NK) cells (28) have already been related to the antitumoral mechanisms of BCG in melanoma.

Although relevant data from the literature have characterized the cellular infiltrate in the TME after BCG treatment, the innate and adaptive immune mechanisms involved in BCG antitumor response against melanoma still not completely understood. In order to explore these immunological mechanisms, we characterized the innate immune responses of B16-F10 murine melanoma cells activated by agonists and used several knockout (KO) mouse strains to perform a screening of molecules that could be involved in the BCG immunotherapy. In addition, we assessed the TME immune profile alterations promoted by BCG in the presence or absence of MyD88 signaling. Finally, we established *in vitro* assays using infected B16-F10 cells co-cultured with macrophages or spleen cells to evaluate MyD88 requirement for cellular activation and cytotoxic activity. In summary, MyD88 signaling showed to be an important pathway on BCG immunotherapy to ensure melanoma control in mice, raising future possibilities for the development of agonists or recombinant BCG to improve cancer immunotherapy.

## 2 Materials and methods

### 2.1 Mouse strains

C57BL/6 wild type (WT) obtained from the Federal University of Minas Gerais (UFMG) animal facility and several knockout (KO) mice were housed in a specific pathogen-free laboratory facility. Mouse deficient for different Toll-like receptors (TLR2^-/-^, TLR3^−/−^, TLR4^−/−^, TLR7^−/−^, TLR3/7/9^−/−^) and MyD88 (MyD88^−/−^) were provided by Dr. Shizuo Akira (Osaka University, Osaka, Japan). Mouse deficient for the interleukin-1 receptor (IL-1R^−/−^) and the double knockout mice for caspase-1 and -11 enzymes (Caspase1/11^−/−^) were provided by Dr. Richard A. Flavell (Yale University School of Medicine, New Haven, USA). Caspase-11 deficient mice (Caspase-11^-/-^) were provided by Dr. Vishva M. Dixit (Genentech Inc., South San Francisco, USA). cGAS and STING deficient mice (cGAS^-/-^, STING^-/-^) were provided by Dr. Glen N. Barber (University of Miami, Miami, USA). Interferon α/β receptor deficient mice (IFNAR^−/−^) were provided by Dr. Dario Zamboni (University of São Paulo, Ribeirão Preto, Brazil). Male and female mice were used at 7-10 weeks of age. Experiments were performed according to protocols that were approved by the Ethics Commission on Animal Use (CEUA) from UFMG under permit #372/2019.

### 2.2 BCG strain

*Mycobacterium bovis* BCG strain Moreau was used in all the experiments *in vitro* and *in vivo* and *Mycobacterium bovis* BCG Pasteur expressing the red fluorescent protein DsRed (BCG-RFP, kindly provided by Dr. André Bafica, UFSC, Florianópolis, Brazil) (29) was used for confocal microscopy. Bacterial stock was generated and stored as previously reported (30).

### 2.3 Tumor cell culture

B16-F10 mouse melanoma parental cell line was kindly provided by Dr. Miriam Teresa Paz Lopes (UFMG, Belo Horizonte, Brazil). B16-F10 cells were grown in DMEM (Gibco) supplemented with 10% heat-inactivated fetal bovine serum - FBS (Gibco), 1% HEPES (Gibco), penicillin G sodium (100 U/ml), streptomycin sulfate (100 μg/ml) and maintained at 37° C in a 5% CO_2_-humidified atmosphere.

### 2.4 Murine melanoma tumor model

B16-F10 melanoma cells (5x10^5^ diluted in 100 μl of PBS) were engrafted subcutaneously into the right flank. Mice were treated with intratumoral injections of BCG (8x10^6^ CFU in 60 μl of PBS) or PBS (mock group) on days 1, 8, and 12 after tumor injection. Tumor volume measurement on days 8, 12, 14 and 18 was assessed using a digital caliper and calculated with the formula: Volume = (length x width^2^)/2. Mice were euthanized at day 18 or earlier if the tumor volume exceeds 1000 mm^3^.

### 2.5 Bone marrow−derived macrophages

Bone marrow cells from C57BL/6 and MyD88^-/-^ mice were obtained by washing the femurs and tibias with cold PBS and the stem cells were cultured in petri dishes in the presence of DMEM (Gibco) with 10% FBS, 1% HEPES, penicillin G sodium (100 U/ml), streptomycin sulfate (100 μg/ml), and 20% L929 cell conditioned medium (LCCM) containing macrophage colony-stimulating factor (M-CSF). The cells were cultured at 37°C in an atmosphere of 5% CO_2_. After four days, 10 ml/dish of complete medium supplemented with LCCM was added into the cells. At day seven of culture, the cells had completely differentiated into bone marrow−derived macrophages (BMDMs) and the macrophages obtained were counted (4x10^5^ or 2x10^5^ cells/well, in case of co-culture) and seeded in 24-well plates for the *in vitro* experiments.

### 2.6 Spleen cells isolation

The spleens obtained from C57BL/6 and MyD88^-/-^ tumor-bearing mice treated with intratumoral injections of BCG or PBS (mock) were macerated, washed with saline and the erythrocytes were lysed with a hemolytic solution (ACK: 155 mM NH_4_Cl, 10 mM KHCO_3_, pH 7.2). After 5 minutes, ACK was neutralized with PBS. Spleen cells were resuspended in complete DMEM medium, filtered using a cell strainer (70µm Nylon - Falcon), counted (4x10^5^ cells/well) and seeded at 24-well plates for the *in vitro* experiments.

### 2.7 B16-F10 co−culture with BMDMs or spleen cells

B16-F10 cells previously seeded in 24-well plates (2x10^5^ cells/well) were infected with BCG (MOI 5). After 24 hours, the supernatant was removed and the cells were washed to remove free BCG. Immune cells in a ratio of 1:1 BMDMs or 1:2 spleen cells were added together with infected or non-infected B16-F10. Cancer cells, BMDMs and spleen cells monocultures were infected with BCG (MOI 5) at the same time as we started the co-culture and used as controls. The cell cultures were maintained for another 24 hours at 37° C in an atmosphere of 5% CO_2_.

### 2.8 *In vitro* innate immune profile evaluation

B16-F10 cells (5x10^5^ cells/well) and BMDMs (5x10^5^ cells/well) were infected with BCG Moreau (MOI 5, 10, 20, and 40) or stimulated with specific agonists such as Pam3CSK4 (1 μg/ml – Invivogen, tlrl-pms), polyI:C (3 μg/ml – InvivoGen, tlrl-pic), ultrapure *Escherichia coli* LPS (100 ng/ml – InvivoGen, tlrl-3pelps), CpG-ODN (1 μg/ml – InvivoGen, tlrl-1826-1), dsDNA90 (3 μg/ml) or *Escherichia coli* LPS (1μg/ml - Sigma-Aldrich, L6529) plus Nigericin (20μM - Sigma-Aldrich, N7143). PolyI:C and dsDNA transfection was performed with Lipofectamine 2000 (3μg/ml – Invitrogen, 11668030) following manufacturer’s instructions. After 24 hours of cell culture, supernatants were collected for ELISA and TRIzol reagent (Invitrogen) was added into the cells to extract RNA. We analyzed TNF-α, IL-6, IL-12, IL-1β, and CXCL10 (IP-10) in the supernatants by ELISA (R&D Systems) according to the manufacturer’s instructions. TRIzol-preserved cells were submitted to qPCR protocol as follow.

### 2.9 Quantitative real−time PCR

B16-F10 and BMDMs stimulated in 24 well-plates as indicated above were homogenized using TRIzol to isolate total RNA accordingly to manufacturer instructions. Cells collected from TME as explained below in section 2.13 were also isolated using TRIzol reagent. Reverse transcription was performed using 2 μg of total RNA in a final volume of 20 μl containing oligo-dT (0.5 μg/μl), dNTP 10 mM, DTT 0.1 M, buffer 5x and reverse-transcriptase (2 U per reaction), using the following cycling parameters: 42°C for 60 min and 70°C for 15 min. The resulting cDNA plus oligo-dT was used as template for qPCR, in a final volume of 10 μL containing SYBR Green PCRMasterMix (Thermo Fischer Scientific, 4309155) and 5 μM of the following primers: β-actin (forward): 5′-GGCTGTATTCCCCTCCATCG-3′; β-actin (reverse): 5′-CCAGTTGGTAACAATGCCATGT-3′; IFN-β (forward): 5′-AGCTCCAAGAAAGGACGAACAT-3′; IFN-β (reverse): 5′-GCCCTGTAGGTGAGGTTGATCT-3’; NOS2 (forward): 5’-AGCACTTTGGGTGACCACCAGGA-3’; NOS2 (reverse): 5’-AGCTAAGTATTAGAGCGGCGGCA-3’; CCR7 (forward): 5’-GGTGGTGGCTCTCCTTGTCATT-3’; CCR7 (reverse): 5’- GCTTTAAAGTTCCGCACGTCCTT-3’; ARG1 (forward): 5’-TGACATCAACACTCCCCTGACAAC-3’; ARG1 (reverse): 5’- GCCTTTTCTTCCTTCCCAGCAG-3’; YM1 (forward): 5’-GGGCATACCTTTATCCTGAG-3’; YM1 (reverse): 5’-CCACTGAAGTCATCCATGTC-3’. Quantitative real-time PCR reaction was performed with QuantStudio 3 Real-Time PCR System (Thermo Fischer Scientific) using the following cycling parameters: 60°C for 10 min, 95°C for 10 min, 40 cycles of 95°C for 15 s, and 60°C for 1 min, and a dissociation stage of 95°C for 15 s, 60°C for 1 min, 95°C for 15 s, and 60°C for 15 s. Data were analyzed using the threshold cycle (ΔΔCt) method and they were presented as relative expression units after normalization to the housekeeping gene (β-actin). PCR measurements were conducted in triplicate.

### 2.10 Intracellular bacterial growth

Colony forming units (CFU) measurement was performed to compare the number of intracellular bacteria in BMDMs and B16-F10 infected with BCG (MOI 5, 10, 20, and 40) in 300 μl/well of DMEM supplemented with 10% FBS and 1% HEPES. Cells were incubated at 37°C in a 5% CO_2_ atmosphere. After 24 hours, cells were washed with saline to remove non-internalized bacteria and lysed with 0.1% saponin (Sigma-Aldrich) per 10-20 minutes at 37° C. Serial dilutions of the lysates in 0.05% Tween80 (Synth, T1029.02.BJ) aqueous solution were plated in Middlebrook 7H11 agar medium (Sigma-Aldrich, M0428-500G) supplemented with 10% oleic acid-albumin-dextrose-catalase (OADC), and the CFUs were counted after 3–4 weeks of incubation at 37°C.

### 2.11 Confocal microscopy

Confocal microscopy was used to confirm BCG internalization. BMDMs or B16-F10 cells were cultured in amount of 1x10^5^ cells/well on poly-D-lysine-coated round coverslips maintained at the bottom of the 24-well plates. Cells were infected with DsRed-expressing bacilli (BCG-RFP). After 24 hrs of culture, supernatants were discarded and the coverslips recovered to start the staining procedure. Cells were fixed with 4% paraformaldehyde (PFA) for 15 min, washed 3 times with PBS and permeabilized with 0.1% Triton in PBS at room temperature (RT). After wash Triton several times, the coverslips were incubated with the conjugated antibody Phalloidin-iFluor 488 (Abcam, ab176753) in a wet chamber for 1 hour at RT. After washing 3 times with PBS, the coverslips were mounted onto glass slides with Fluoromount G containing DAPI (Invitrogen, 00495952). Images were taken at the Center for Gastrointestinal Biology (UFMG, Belo Horizonte, Brazil) with Nikon Eclipse Ti with a A1R confocal head equipped with four different lasers (excitation at four wavelengths: 405, 488, 546, and 647 nm) and emission bandpass filters at 450/50, 515/30, 584/50, and 663/738 nm. Objective Plan Apo 20x. 3D rendering and image analyses was performed by using Volocity 6.2 (PerkinElmer) software.

### 2.12 Cell viability assay

BMDMs or B16-F10 cells (5x10^5^ cells/well) were seeded in 24-well plates and infected with BCG (MOI 5, 10, 20, and 40) in a final volume of 300 µl. Cells were cultured for 24 h at 37°C in a 5% CO2 atmosphere. Briefly, BMDMs and B16-F10 cells infected or not infected were incubated with 1% EDTA saline cold solution (Gibco) for 5-10 minutes. Afterwards, cell suspension was counted using 0.02% Trypan blue in PBS and a Neubauer chamber to determine the number of viable (unstained) cells. Cell viability of not infected controls for each cell line were normalized to 100%.

### 2.13 Cellular infiltrate evaluation in TME by flow cytometry

WT and MyD88^-/-^ mice were euthanized 18 days after tumor cells injection and BCG treatment. Tumors were dissected and the cells dissociated with collagenase IV (200 U/ml) as previously shown (26). The cell suspension was filtered using cell strainer (70 µm) and viability was checked by Trypan blue dye exclusion method. TME cells (1x10^6^ cells/well) were analyzed by flow cytometry. Different antibodies (anti-mouse) were used for evaluation of myeloid cells, lymphocytes and IFN-γ cytokine production by lymphocytes. Briefly, cells were incubated for 20 min with anti-mouse CD16/32 to block Fc receptors (BD Bioscience) in FACS buffer (PBS, 0.25% BSA, 1 mM NaN_3_) and were stained for surface markers. For IFN-γ staining, cells were previously incubated with Brefeldin A (1 μg/well; Sigma-Aldrich) during 4 h at 37°C, 5% CO2. The following conjugated antibodies were used: anti-CD11c FITC (clone HL3; BD Bioscience); anti-CD11b APC-Cy-7 (clone M1/70; BD Bioscience); anti-F4/80 PerCP-Cy5.5 (clone T45-2342; BD Bioscience); anti-Ly6G PE (clone 1A8; BD Bioscience); anti-CD3 PE-Cy-7 (clone 145-2C11; BD Bioscience); anti-CD4 APC-Cy-7 (clone GK1.5; BD Bioscience); anti-CD8 FITC (clone 53-6.7; BD Bioscience); anti-NK1.1 PE (clone PK136; BD Bioscience); anti–IFN-γ APC (clone XMG1.2; BD Bioscience). The appropriate isotype controls were used. Attune Acoustic Focusing Cytometer (Life Technologies, Carlsbad, USA) was used to collect more than 100,000 events and data were analyzed using FlowJo Software (Tree Star, Ashland, USA). Gating strategy is shown in **Supplementary Figure 1**. Average viability of experiments is 54-79% assessed by Zombie NIR Fixable Viability Kit (Biolegend, California; data not shown).

### 2.14 Killing assay

To perform the killing assay, 2x10^7^ B16-F10 cells were resuspended in 1ml of PBS and labeled with 2,5uM CellTrace carboxyfluorescein succinimidyl ester (CFSE) solution (ThermoFisher, C34554) for 20 min according to the manufacturer’s instructions. B16-F10 (1x10^5^ cells/well) resuspended in DMEM medium were seeded in 24-well plate, infected with BCG (MOI 5) and cultured at 37°C in an atmosphere of 5% CO_2_. After 24 hrs of infection, the spleen cells from tumor-bearing C57BL/6 or MyD88^-/-^ were co-cultured with previously CFSE-stained B16-F10 cells in different ratios (1:10 and 1:20 tumor/spleen cells) for another 24 hrs. Tumor and immune cells were recovered using 1% EDTA solution for 10 min. The total cells from each 24 well were transferred to a 96-well plate. Killed and viable cells were evaluated by flow cytometry, where CFSE positive staining indicate the live tumor cells. Attune Acoustic Focusing Cytometer (Life Technologies) was used to collect more than 100,000 events and data were analyzed using FlowJo Software (Tree Star, Ashland, USA). Gating strategy is shown in **Supplementary Figure 2**.

### 2.15 Statistical analysis

Results are presented as the mean ± SD. Statistically significant differences among the results obtained were evaluated by 2-way ANOVA followed by the Bonferroni *post hoc* test (P < 0.05), one-way ANOVA followed by the Tukey *post hoc* test (P < 0.05) or the Student t-test (P < 0.05) as stated in figure legends. Statistical analysis was performed using GraphPad Prism 8.4.3 (GraphPad Software, San Diego, USA).

## 3 Results

### 3.1 BCG immunotherapy efficiently controls B16-F10 melanoma tumors

**Figure 1:**
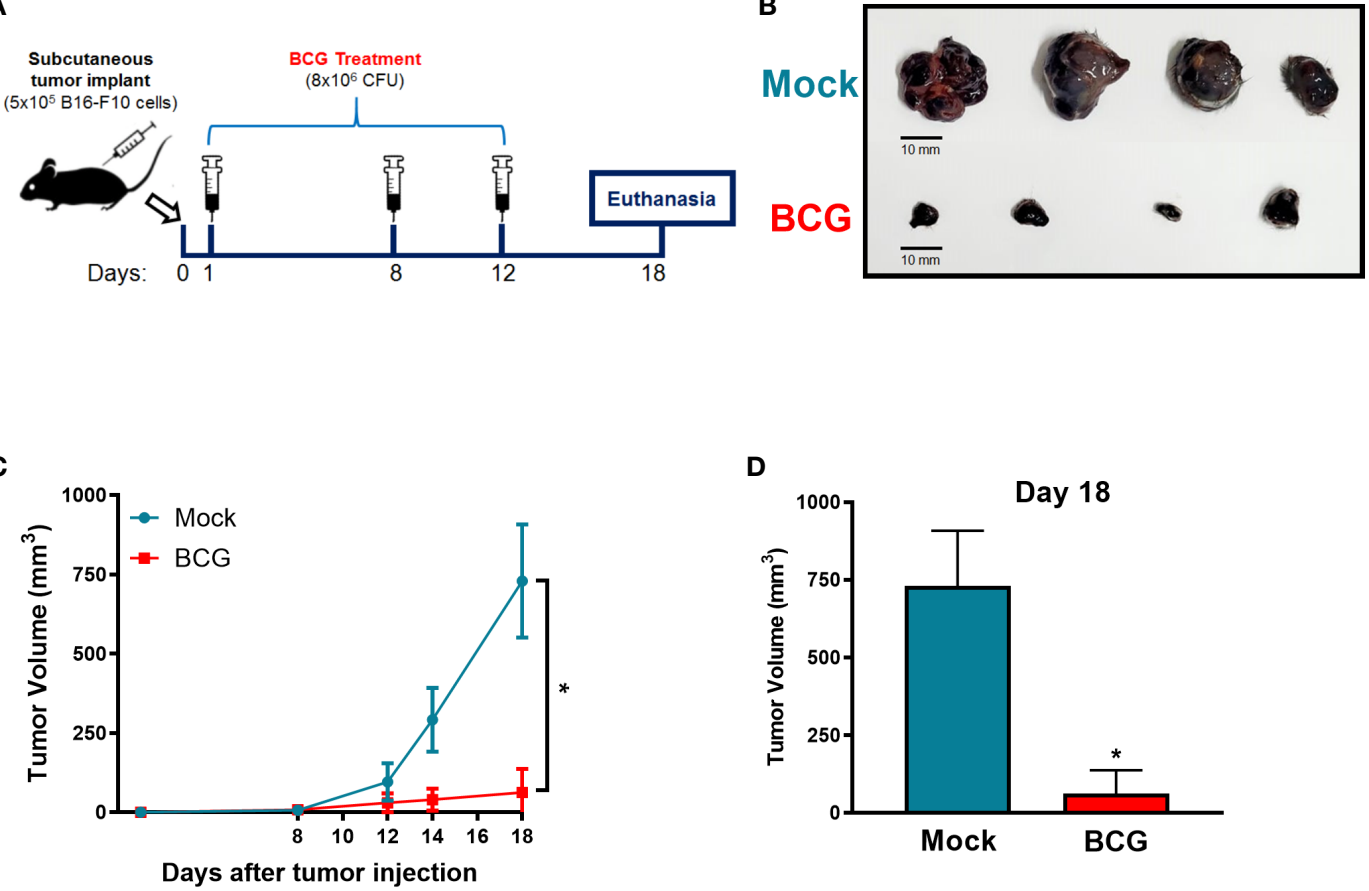
BCG treatment controls subcutaneous melanoma (B16-F10) tumor growth. **(A)** Graphical scheme of the established subcutaneous murine melanoma model treated with BCG. 5×10^5^ B16-F10 cells was engrafted by subcutaneous injection into the right flank of C57BL/6 mouse. BCG (8×10^6^ CFU) or PBS (Mock) treatments were realized on days 1, 8 and 12 post-tumor injection. Tumor growth was monitored with a digital caliper in different time points until day 18. **(B)** After 18 days, mice were euthanized and tumors dissected to evaluate BCG treatment effect to control tumor development compared with Mock treated (scale bar, 10 mm). Tumor growth curves **(C)** and the final tumor volumes after 18 days **(D)** are shown. **(C, D)** represent the tumor volumes mean and standard deviation summary of C57BL/6 WT (Mock: n=14; BCG: n=20) control mice pooled of all the following *in vivo* experiments comparing WT and KO mice. *Statistically significant compared to mock (ANOVA; P ≤ 0.0001).

First, we designed an experimental model of tumor control in response to BCG treatment, which allows reliability to compare the effect of BCG in WT and in different KO mice. Intratumorally BCG injection is the best route of administration (31) and starting the treatment one day after tumor implantation has been described as the optimal timing for tumor control (32). Therefore, we established a subcutaneous melanoma model using B16-F10 cells (5x10^5^) treated intratumorally with BCG (8x10^6^ CFU) or PBS (mock) on days 1, 8 and 12 after tumor injection (**Figure 1A**), like we described for MB49 bladder cancer model in our previous study (26). Tumor growth was monitored in different time points until day 18, when we euthanized the animals, dissected the tumors and observed the efficiency of BCG treated mice to control the development of B16-F10 tumors (**Figure 1B**). BCG treatment robustly reduced tumor growth in all mice analyzed (**Figures 1C, D**), reinforcing the efficacy of this immunotherapy strategy to treat melanoma and providing a reliable model to investigate the role of different immune mechanisms using WT and KO mice.

### 3.2 Innate immune profile of B16-F10 in response to BCG and other agonists

**Figure 2:**
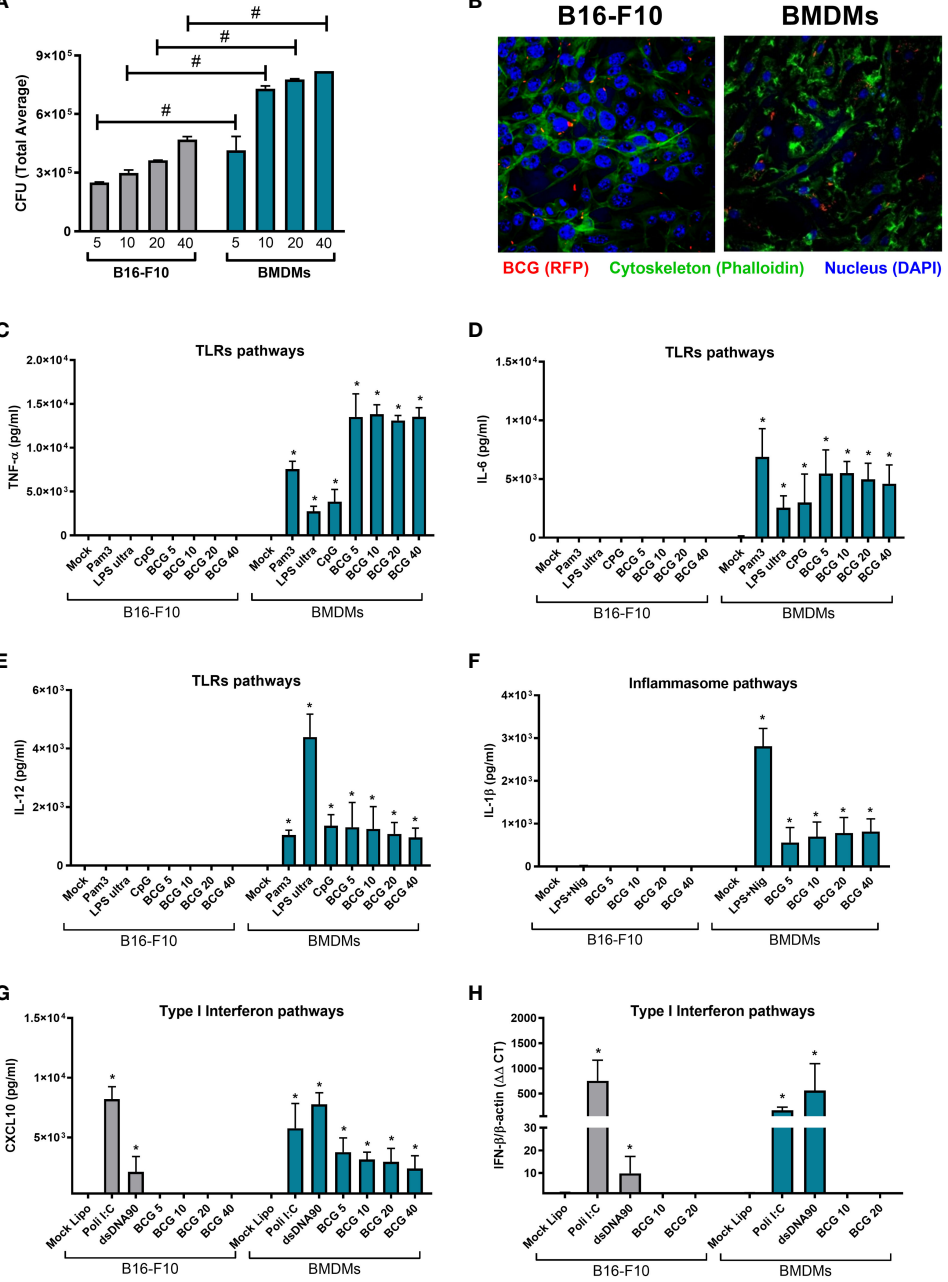
Innate immune profile of B16-F10 in response to BCG infection and other innate immune agonists. **(A)** B16-F10 cells and BMDMs were infected *in vitro* with BCG (MOI 5, 10, 20 and 40) for 24h. After this period, the cell cultures were washed twice with PBS to remove free bacteria and subsequently lysed, diluted and plated for quantification of the intracellular bacterial load by counting total CFU after 21-30 days. **(B)** Confocal microscopy images comparing the intracellular localization of BCG Pasteur expressing RFP (red) (MOI 5) in BMDMs or B16-F10. Cell cytoskeleton was stained with Phalloidin (green) and nucleus with DAPI (blue). **(C–E)** B16-F10 cells and BMDMs were infected with BCG (MOI 5, 10, 20 and 40) or stimulated with TLRs agonists, like Pam3CSK4 (1μg/ml), ultrapure LPS (100 ng/ml), and CpG 1826 (1 μg/ml) for 24 hrs; or **(F)** with E. coli LPS for 4 hrs plus Nigericin (20μM) in the final 45 minutes of incubation to activate the Inflammasome; or **(G, H)** transfected with Poly I:C (3 μg/ml) or dsDNA90 (3 μg/ml) in addition to Lipofectamine 2000 (3ug/ml) to evaluate the induction of type I IFN pathways. The production of TNF-α **(C)**, IL-6 **(D)**, IL-12 **(E)**, IL-1β **(F)** and CXCL10 **(G)** in cell cultures supernatants were analyzed after 24 h of stimulus by ELISA. IFN-β expression after 1 h was determined by qPCR **(H)**. All qPCR results were relative to β-actin mRNA as a normalizer. B16-F10 unstimulated (Mock) or BMDM (Mock) were used as control. The values are representative of at least three independent experiments. *Statistically significant compared to mock from the same cell line (ANOVA; P ≤ 0.05). ^#^Statistically significant comparing B16-F10 and BMDMs with the same treatment (ANOVA; P ≤ 0.05).

The susceptibility of different tumor cell lines to BCG infection has already been described by another group (33) and in our previous work we confirmed the permissiveness of MB49 bladder cancer cell to BCG infection, although MB49 cells were unable to activate an inflammatory response to BCG (26). In the present study, we evaluated the ability of BCG to infect B16-F10 melanoma cells by quantifying the intracellular bacterial load through CFU (**Figure 2A**) and using confocal images to confirm the internalization of the BCG (RFP) (**Figure 2B**; **Supplementary Video S1**; **Supplementary Video S2**). We observed a lower amount of bacteria internalized by B16-F10 cells (**Figures 2A, B**; **Supplementary Video S1**) when compared to professional phagocytic cells (BMDMs) (**Figures 2A, B**; **Supplementary Video S2**), but the intracellular bacterial load variation occurs in the same logarithmic scale (**Supplementary Figure 3A**). We also compared the infection with different BCG MOI (5, 10, 20 and 40) and observed that MOI 5 did not affect B16-F10 or BMDMs cell viability (**Supplementary Figure 3B**).

After confirming that BCG is able to infect B16-F10 cells, we decided to investigate whether BCG would be capable to elicit important innate immune pathways in tumor cells. We evaluated the activation of TLRs, inflammasome, and type I interferon pathways related to the BCG-induced antitumor response. Concerning the TLRs pathways, the stimulus with different BCG loads (MOI 5, 10, 20, and 40) or with the specific agonists for TLR2 (Pam3CSK4), TLR4 (ultrapure LPS), or TLR9 (CpG 1826) were not able to induce B16-F10 cells to secrete inflammatory cytokines such as TNF-a (**Figure 2C**), IL-6 (**Figure 2D**) or IL-12 (**Figure 2E**). In terms of inflammasome activation, we evaluated IL-1β secretion in response to BCG or a positive control (*Escherichia coli* LPS plus Nigericin) and we observed that B16-F10 was not able to secrete IL-1β (**Figure 2F**). In contrast, the same stimuli promoted a strong cytokine response in BMDMs, our control group that represents an immune cell with functional innate immune pathways (**Figures 2C–F**). Finally, we observed that BCG infection induced moderate levels of CXCL10 secretion from BMDMs (**Figure 2G**) and was unable to stimulate B16-F10 cells or BMDMs to express detectable *IFN-β* mRNA (**Figure 2H**). However, type I interferon pathway was strongly activated in B16-F10 when we used specific agonists to TLR3 (Poly I:C) or cGAS-STING (dsDNA90) (**Figures 2G, H**). These supports our previous findings with MB49 and BMDMs (26) suggesting that immune cells, not tumor cells, are essential for the immune response to BCG.

### 3.3 Innate immune pathways involved in melanoma (B16-F10) treatment with BCG

**Figure 3:**
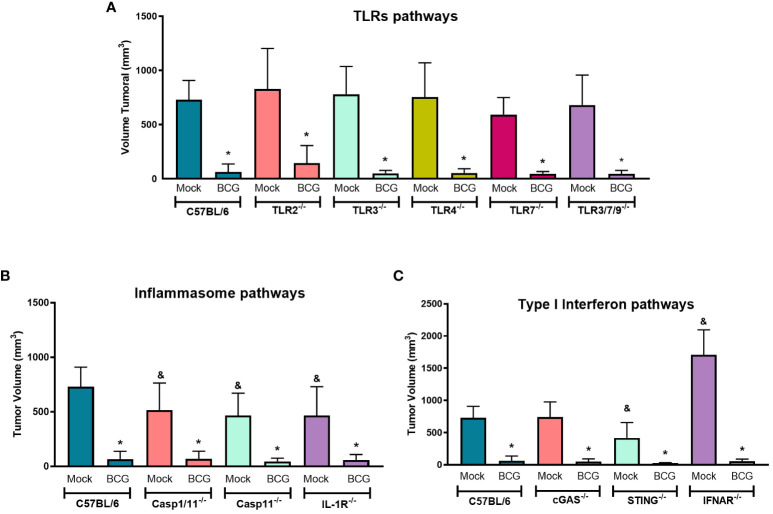
TLRs, inflammasome and type I IFN pathways are not directly involved with BCG-elicited melanoma control. C57BL/6 WT (Mock: n=14; BCG: n=20) and different KO mice were submitted to the subcutaneous melanoma model injection and subsequent treatment with PBS (Mock) or BCG as previously explained in **Figure 1A**. **(A)** TLRs pathways evaluated using TLR2^-/-^ (Mock: n=14; BCG: n=27), TLR3^-/-^ (Mock: n=6; BCG: n=10), TLR4^-/-^ (Mock: n=10; BCG: n=8), TLR7^-/-^ (Mock: n=7; BCG: n=9), and TLR3/7/9^-/-^ (Mock: n=8; BCG: n=10) deficient mice. **(B)** Inflammasome and IL-1 signaling pathways were investigated using Caspase-1/11^-/-^ (Mock: n=9; BCG: n=9), Caspase-11^-/-^ (Mock: n=12; BCG: n=12), and IL-1R^-/-^ (Mock: n=11; BCG: n=11) deficient mice. **(C)** cGAS-STING and type I IFN signaling pathways were evaluated using cGAS^-/-^ (Mock: n=5; BCG: n=8), STING^-/-^ (Mock: n=13; BCG: n=12), and IFNAR^-/-^ (Mock: n=6; BCG: n=8) deficient mice. The final tumor volumes (day 18) comparing C57BL/6 WT with deficient mice are represented in mean and standard deviation from the results of at least two independent experiments. C57BL/6 WT data represents the summary of control animals from all *in vivo* experiments comparing WT and KO mice. *Statistically significant compared to the respective untreated (Mock) control (ANOVA; P ≤ 0.05). ^&^Statistically significant compared to the untreated (Mock) C57BL/6 WT group (ANOVA; P ≤ 0.05).

In order to determine the role of innate immune pathways related to BCG immunotherapy *in vivo*, we compared our model in WT (**Figure 1A**) with different KO mice (TLR2^-/-^, TLR3^-/-^, TLR4^-/-^, TLR7^-/-^, TLR3/7/9^-/-^, Caspase-1/11^-/-^, Caspase-11^-/-^, IL-1R^-/-^, cGAS^-/-^, STING^-/-^ and IFNAR^-/-^). BCG treatment showed a similar response to control tumors in WT and all KO mice tested, suggesting that the antitumor response induced by BCG is not direct dependent on single TLRs (**Figure 3A**), inflammasome and IL-1 signaling (**Figure 3B**) or cGAS-STING and type I interferon pathways (**Figure 3C**). It is interesting to note that B16-F10 tumors (Mock treated) grow less in Caspase-1/11^-/-^, Caspase-11^-/-^ and IL-1R^-/-^ (**Figure 3B**), which confirms another study that showed the importance of IL-1β signaling in B16 melanoma tumor progression (34). Moreover, the antitumor effect of type I IFN signaling is well described and B16-F10 tumors grafted on IFNAR^-/-^ presented an increased tumor development compared to WT (35) as we also demonstrated in **Figure 3C**. Therefore, we could not identify the impact of a single innate immune pathway involved in tumor regression induced by BCG immunotherapy, but these pathways could be acting in a coordinated manner to control melanoma in response to BCG.

### 3.4 BCG depends on MyD88 signaling for an effective antitumor response

**Figure 4:**
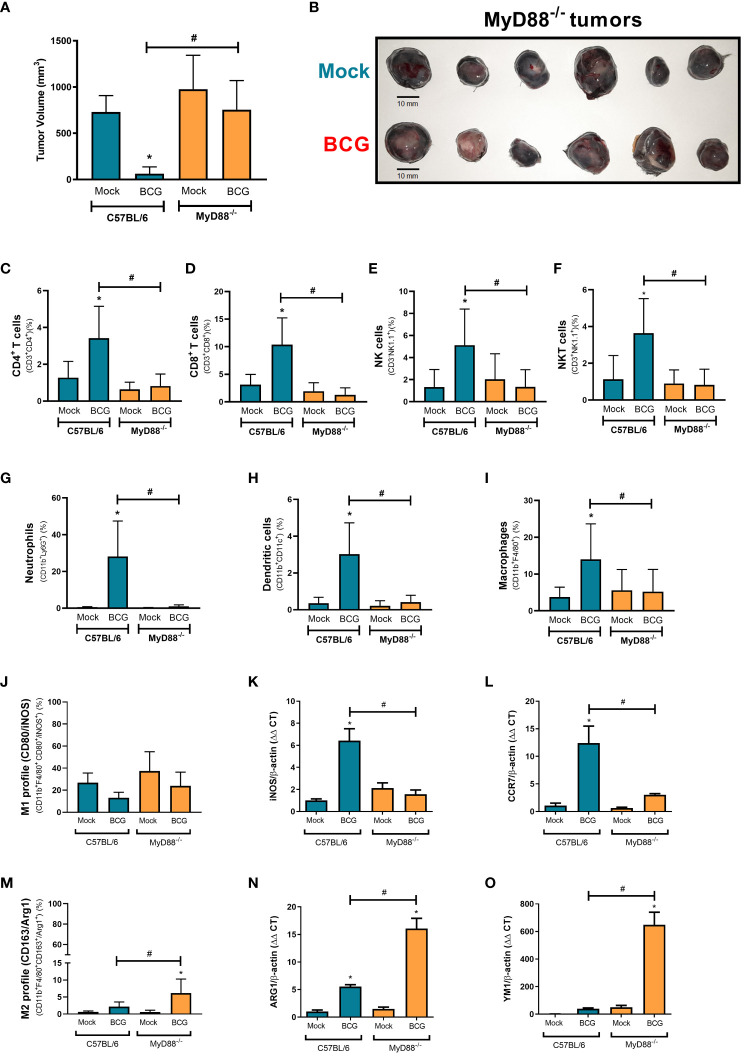
MyD88-dependent BCG immunotherapy modulates the infiltration of effective anti-tumor immune cells into the TME. C57BL/6 WT and MyD88^-/-^ deficient mice were submitted to the subcutaneous melanoma model injection and subsequent treatment with PBS (Mock) or BCG as previously explained in **Figure 1A**. **(A)** The final tumor volumes (day 18) comparing C57BL/6 WT (Mock: n=14; BCG: n=20) with MyD88^-/-^ (Mock: n=17; BCG: n=21) are represented in mean and standard deviation from the results of at least three independent experiments. **(B)** After 18 days, mice were euthanized and MyD88^-/-^ tumors dissected to evaluate BCG treatment effect represented in the image (scale bar, 10 mm). **(C–I)** C57BL/6 WT and MyD88^-/-^ tumors were also dissected on day 18 and the cells were dissociated with collagenase IV to evaluate cell infiltrate by flow cytometry using specific markers to: CD4^+^ T cells (CD3^+^ CD4^+^) **(C)**; CD8^+^ T cells (CD3^+^ CD8^+^) **(D)**; natural Killer cells (CD3^-^NK1.1^+^) **(E)**; NKT cells (CD3^+^NK1.1^+^) **(F)**; neutrophils (CD11b^+^Ly6G^+^) **(G)**; dendritic cells (CD11b^+^ CD11c^+^) **(H)**; macrophages (CD11b^+^ F4/80^+^) **(I)**; M1-like macrophages (CD11b^+^ F4/80^+^ CD80^+^/iNOS^+^) **(J)**; and M2-like macrophages (CD11b^+^ F4/80^+^ CD163^+^/ARG1^+^) **(M)**. In order to confirm macrophages profile, we evaluate by qPCR the following mRNA expression in the TME: iNOS **(K)** and CCR7 **(L)** for M1-like macrophages; ARG1 **(N)** and YM1 **(O)** for M2-like macrophages. All qPCR results were relative to β-actin mRNA as a normalizer. C57BL/6 WT untreated (Mock) were used as control. Graphs represent the cell percentage relative to total cell population contained in TME. The results are representative of at least three independent experiments. *Statistically significant compared to the respective untreated control (ANOVA; P ≤ 0.05). ^#^Statistically significant comparing C57BL/6 WT and MyD88^−/−^ treated with BCG (ANOVA; P ≤ 0.05).

The majority of the innate immune receptors investigated in **Figure 2** and **Figure 3**, such as IL-1R and TLRs - except TLR3, rely on MyD88 recruitment to signal through specific intracellular cascades. MyD88 affects several innate immune pathways concomitantly. Thus, we decided to investigate the relevance of MyD88 for BCG treatment against B16-F10 tumors. BCG treatment was not able to control melanoma growth in MyD88^-/-^ mice that presented similar volume as the untreated tumors (**Figures 4A, B**), differing from BCG response in WT mice. BCG is important to recruit antitumor immune cells to the TME (26). Then, we performed a flow cytometry assay to evaluate the immune cells infiltrated in melanoma from WT and MyD88^-/-^. The intratumoral treatment with BCG in WT mice promoted the increment of several immune cells in the TME, such as CD4^+^ T lymphocytes (**Figure 4C**), CD8^+^ T lymphocytes (**Figure 4D**), NK (**Figure 4E**), NKT cells (**Figure 4F**), neutrophils (**Figure 4G**), dendritic cells (**Figure 4H**), and macrophages (**Figure 4I**). In addition, we performed flow cytometry and qPCR analyses to evaluate the macrophages profile in the TME from WT and MyD88^-/-^ mice. BCG treatment induces an inflammatory profile of M1-like macrophages (CD80, iNOS and CCR7 markers) in WT tumors, while in MyD88^-/-^ we observed a different profile with an increase in M2-like macrophages (CD163, ARG1 and YM1 markers) (**Figures 4J–O**). Taken together with the immune cells infiltrated in the TME, the M1-like macrophages polarization only in WT reinforce the importance of MyD88 signaling for the recruitment of inflammatory cells leading to the efficient melanoma growth control.

### 3.5 Innate and adaptive immune responses to BCG are activated in a MyD88-dependent manner

**Figure 5:**
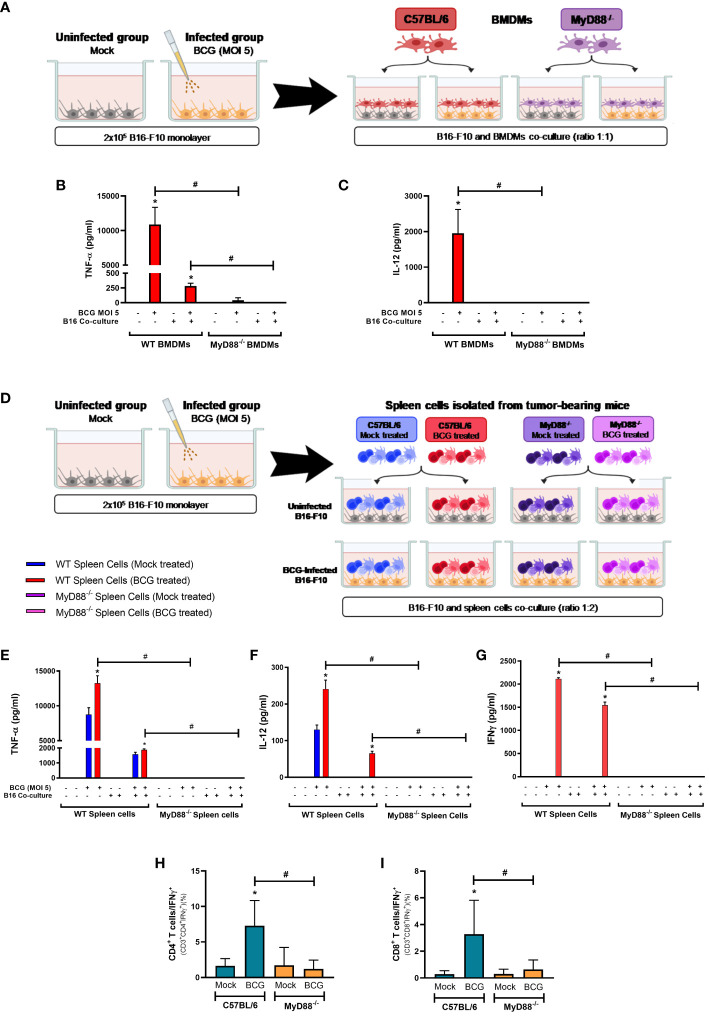
MyD88 signaling impacts BCG-elicited cytokine response in co-cultures with B16-F10 and BMDMs or spleen cells and in the TME. Schemes of B16-F10 co-cultured with BMDMs **(A)** or spleen cells **(D)** derived from WT and MyD88^-/-^ mice. **(A–C)** B16-F10 cells (2x10^5^ cells/well) were previously infected with BCG (MOI 5) for 24 h. After this period, cell cultures were washed twice with PBS to remove free BCG in the supernatant, previously to the addition of C57BL/6 WT or MyD88^−/−^ BMDMs in the co-culture (tumor/BMDMs ratio 1:1). After 24h, the production of TNF-α **(B)** and IL-12 **(C)** were analyzed by ELISA. B16-F10 and BMDMs alone were also infected with BCG (MOI 5) and used as controls. **(D–G)** B16-F10 cells (2x10^5^ cells/well) were previously infected with BCG (MOI 5) for 24 h. After this period, cell cultures were washed twice with PBS to remove free BCG in the supernatant. Following, spleen cells isolated from tumor-bearing WT and MyD88^-/-^ mice treated with PBS (Mock treated groups) or BCG (BCG treated groups) were added in the co-culture (tumor/spleen cells ratio 1:2). After 24h, the production of TNF-α **(E)**, IL-12 **(F)** and IFN-γ **(G)** were analyzed by ELISA. B16-F10 and spleen cells alone were also infected with BCG (MOI 5) and used as controls. **(H, I)** C57BL/6 WT and MyD88^-/-^ deficient mice were submitted to the subcutaneous melanoma model injection and subsequent treatment with PBS (Mock) or BCG as previously explained in **Figure 1A**. After 18 days of tumor injection, mice were euthanized, tumors dissected and cells were dissociated with collagenase IV to evaluate IFN-γ production by lymphocytes in the TME, using flow cytometry with specific markers to: CD3^+^CD4^+^IFN-γ^+^ **(H)** and CD3^+^CD8^+^IFN-γ^+^ **(I)**. Graphs represent the cell percentage relative to total cell population contained in TME. The values are representative of at least three independent experiments. *Statistically significant compared to the respective untreated control (monoculture or co-culture) (ANOVA; P ≤ 0.05). ^#^Statistically significant comparing the same stimulus in WT and MyD88^-/-^ (ANOVA; P ≤ 0.05). The schemes **(A, D)** were created with BioRender.com.

In order to confirm the relevance of MyD88 signaling for BCG immunotherapy against melanoma, we developed a co-culture strategy simulating the interactions that probably occurs between BCG-infected tumor cells and the infiltrated immune cells in the TME. We infected B16-F10 cells with BCG (MOI 5) and after 24 hours these cells were co-cultured with BMDMs from WT or MyD88^-/-^ mice for another 24 hours (**Figure 5A**). BMDMs infected with BCG or co-cultured with infected B16-F10 cells produced inflammatory cytokines such as TNF-α (**Figure 5B**) or IL-12 (**Figure 5C**) in a MyD88-dependent manner. We also co-cultured infected B16-F10 with spleen cells isolated from tumor-bearing WT or MyD88^-/-^ animals previously treated with BCG or untreated (**Figure 5D**). Once again, the results showed the importance of MyD88 to induce an immune response demonstrated by the increased production of TNF-α (**Figure 5E**), IL-12 (**Figure 5F**) and IFN-γ (**Figure 5G**) in co-cultures with different WT spleen cells, but not with MyD88^-/-^ splenocytes. Interestingly, the spleen cells isolated from tumor-bearing WT mice previously treated with BCG presented a strong cytokine production when compared with the spleen cells from mock treated-mice (**Figures 5E, F**). Accordingly, these results suggest that MyD88 signaling is crucial for BCG to promote immune response in BMDMs and for priming spleen cells.

**Figure 6:**
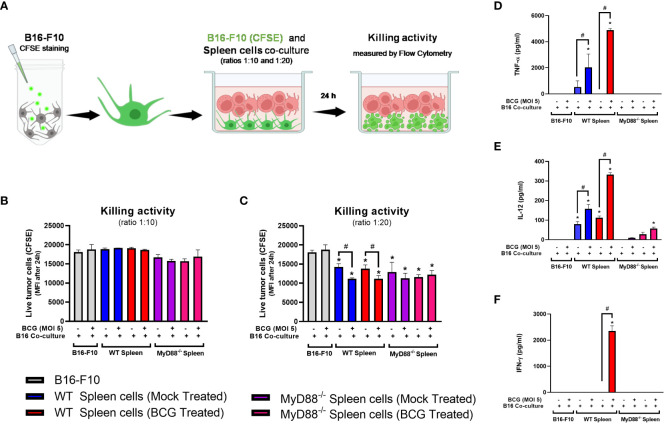
WT spleen cells showed an increment in killing activity when co-cultured with B16-F10 infected with BCG. **(A)** Scheme of the Killing assay representing the CFSE-stained B16-F10 cells co-cultured with spleen cells in different target ratios (tumor/spleen cell ratios 1:10 and 1:20) and the cytotoxic activity measured by flow cytometry after 24h. **(B, C)** Killing activity of CFSE-stained B16-F10 cells co-cultured with spleen cells isolated from tumor-bearing WT and MyD88^-/-^ mice previously treated with PBS (Mock treated groups) or BCG (BCG treated groups) in target ratios of 1:10 **(B)** or 1:20 **(C)**, indicated by the reduction Mean Fluorescence Intensity (MFI) of the CFSE staining. The production of TNF-α **(D)**, IL-12 **(E)** and IFN-γ **(F)** in the cell cultures were analyzed by ELISA after 24h. The results are representative of at least three independent experiments. *Statistically significant compared to the respective untreated control (ANOVA; P ≤ 0.05). ^#^Statistically significant comparing C57BL/6 WT and KO treated with BCG (ANOVA; P ≤ 0.05). ^&^Statistically significant comparing the same spleen cells group co-cultured with B16-F10 (CFSE) infected or not with BCG (T-Test; P ≤ 0.05). The scheme **(A)** was created with BioRender.com.

**Figure 7:**
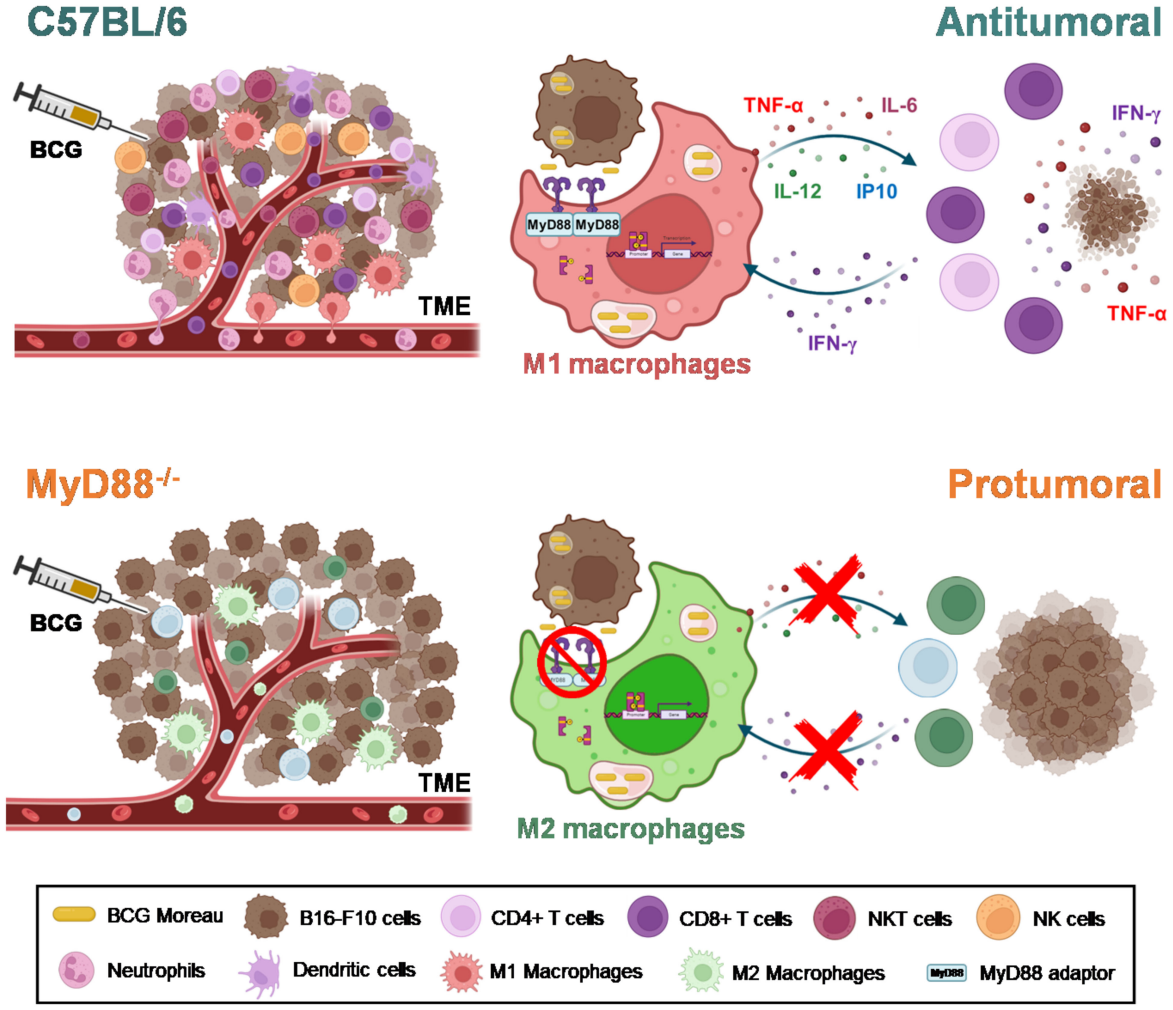
MyD88 impacts on both innate and adaptive responses to BCG, culminating in the efficient antitumor effect controlling melanoma growth. Graphical abstract represents the impact of MyD88 signaling in TME infiltrate modulated by BCG treatment in B16-F10 tumors, as well as in BCG-stimulation of macrophage and lymphocytes. The intratumoral treatment with BCG in C57BL/6 tumors induces the recruitment of a robust infiltrate of inflammatory cells (M1-like macrophages, neutrophils, dendritic cells, CD4^+^ and CD8^+^ T cells, NK and NKT cells), polarizing the TME to a “hot” profile (antitumoral) associated with tumor control. The adaptor molecule MyD88 probably acts as a bridge between the innate and the adaptive immunity in response to BCG immunotherapy. The Graphical abstract was created with BioRender.com.

BCG is well known as a Type1 T helper (Th1) response-driving agent. In order to evaluate the impact of MyD88 on IFN-γ production by T lymphocytes, we analyzed this cytokine in the TME by flow cytometry. The increase in IFN-γ production by CD4^+^ (**Figure 5H**) and CD8^+^ T cells (**Figure 5I**) in the TME were not detected in cells from MyD88^-/-^ animals. Interestingly, only the spleen cells isolated from tumor-bearing WT mice previously treated with BCG and restimulated *in vitro* with BCG were capable to produce IFN-γ, that was abrogated in the absence of MyD88. Therefore, we decided to investigate the killing activity of WT or MyD88^-/-^ spleen cells in co-culture with B16-F10 previously treated with CFSE as target cells (**Figure 6A**). We observed that despite the important role of MyD88 to activate the innate and adaptive immune cells in response to BCG, there was no difference between WT and MyD88^-/-^ splenocytes derived from animals treated or not with BCG to kill B16-F10 target cells in 1:10 or 1:20 ratio (**Figures 6B, C**). Finally, an important observation is that spleen cells from WT mice showed an increment in killing activity when they were co-cultured with B16-F10 infected with BCG, profile not observed in the co-culture with MyD88^-/-^ splenocytes. Inflammatory cytokines TNF-α, IL-12 and IFNγ (**Figures 6D–F**) were increased especially in cell cultures with spleen cells from WT mice treated *in vitro* with BCG, but not with spleen cells from MyD88^-/-^, reinforcing the importance of MyD88 to induce cell death mechanisms and tumor control. Taken together, our data summarized in the graphical abstract (**Figure 7**) demonstrate that MyD88 molecule is crucial to induce an efficient BCG-antitumor response to control murine melanoma.

## 4 Discussion

The relevance of innate immune pathways in melanoma treatment is highlighted by some immunotherapy approaches such as TLR7 in the adjuvant therapy with imiquimod (36), TLR9 in the development of the emerging agonists tilsotolimod and vidutolimod (37), STING in the case of ADU S-100 (38), and the clinical use of IFN-α (39). The activation of several innate immune pathways in both tumor cells and other immune cells present in the TME is essential to provide an efficient antitumor response. It is already known that many tumor cell lines have deficiencies in some innate immune pathways, which can alter the response to immunotherapy (40). Here, we first explored the immunological profile of B16-F10 melanoma cells in response to BCG and other specific agonists. B16-F10 did not respond to BCG stimulation and some innate immune pathways appear to be non-functional in these cells such as TLR2, TLR4, TLR9, and inflammasomes. Despite that, B16-F10 still able to produce CXCL10 and IFN-β in response to TLR3 or cGAS-STING agonist. There is a promising benefit in combining BCG with poly I:C (41) or engineering a recombinant BCG strain to overexpress cyclic di-AMP (25) to treat melanoma. However, TLR3 (42), cGAS and STING (40) are differentially expressed in human melanoma cell lines. Therefore, further studies could help to develop new therapies associating BCG and other agonists with the purpose to improve immunotherapy against melanoma.

The immune response elicited by BCG is broadly described as involving TLR2, TLR4, TLR9, and NOD2. In this study, we showed that MyD88 is an important molecule in BCG-mediated antitumor response against melanoma. In the absence of MyD88 signaling, several innate immune pathways are affected, such as the majority of TLRs (except TLR3), IL-1R, IL-18R, IL-33R/ST2, and IL-36R (17). Other receptors independent of the TIR domain also rely on MyD88, like RAGE (43) and TACI (44). Our data demonstrate that the role of TLR2, TLR3, TLR4, TLR7, TLR9, and IL-1R are not relevant for BCG treatment in B16-F10 mouse model. The involvement of other MyD88-dependent receptors will be the subject of future investigation. However, it is most likely that MyD88 signaling elicited by BCG treatment could involve the activation of several pattern recognition receptors concomitantly.

Our data suggests that the main role of MyD88 signaling in BCG-elicited antitumor response is to promote the recruitment and activation of antitumor immune cells into the TME as well as establishing an interplay between the innate and adaptive immune response through priming immune cells. Herein, we did not observed macrophages, neutrophils, dendritic cells, CD4^+^ and CD8^+^ T lymphocytes, NKT and NK cells subsets recruited into TME after BCG treatment in the MyD88-deficiency animals. Other investigators have claimed that BCG-mediated tumor immunity is dependent on macrophages and dendritic cells (DCs) in the melanoma model (7). We also detected a complete abrogation of TNF-α and IL-12 production in MyD88^-/-^ BMDMs and spleen cells in response to BCG infection. In our previous study using MB49 tumor, we also demonstrated that inflammatory macrophages (M1) is an important cell subpopulation present in TME of BCG treated animals (26). In addition to previous studies, our current results explore the interplay in innate and adaptive immunity, reinforcing the important role of MyD88 for T cell-mediated IFN-γ production. The production of IFN-γ was previously described as the BCG-elicited effector function of tumor-specific CD4^+^ T cells in subcutaneous MB49 bladder tumors (45). The adaptive–innate immune interactions involving MyD88-dependent polarization of a Th1 response and subsequent IFN-γ release was recently described as a mechanism induced by BCG vaccination to promote protection against SARS-CoV2 (46). BCG heterologous immunological memory has been also related as a possible mechanism involved in cancer control (47), but the role of MyD88 in trained immunity and cancer is still an important scientific gap to be addressed.

In summary, our data brings insight in the importance of BCG immunotherapy against melanoma, demonstrating that the intratumoral treatment with BCG in C57BL/6 mice induces the recruitment of a robust inflammatory cells infiltrate (M1-like tumor-associated macrophages, neutrophils, dendritic cells, CD4^+^ and CD8^+^ T cells, NK and NKT cells) and polarize the TME to a “hot” profile (antitumoral) associated with tumor control. In this regard, the *in vitro* data suggest that the free bacteria or the infected tumor cells present into the TME are capable to stimulate the activation of macrophages and the release of cytokines and chemokines (TNF-α, IL-6, IL-12, and CXCL10) in a MyD88-dependent manner. The adaptor molecule MyD88 probably acts as a bridge between the innate and the adaptive immunity, by promoting the activation of antigen presenting cells and consequently priming lymphocytes that exhibit IFN-γ secretion and cytotoxicity profile against tumor cells. In contrast, tumors from MyD88^-/-^ animals treated with BCG do not show an increase in the inflammatory infiltrate and induces an M2-like tumor-associated macrophages profile, indicating a “cold” pattern of the TME (protumoral) favoring tumor growth. In the absence of MyD88 signaling, the activation of the pro-inflammatory innate immunity cells (such as M1-like macrophages) does not occur, the cytokine production is abrogated, the response is not polarized to a Th1 profile and the tumor cells are allowed to proliferate (**Figure 7**). Targeting M1-like macrophages polarization is a potential therapeutic approach (48) induced by BCG immunotherapy previously showed in murine bladder cancer (26, 49) and also showed for the first time in the present study in controlling melanoma growth. Taken together, our results demonstrate the fundamental role of MyD88 in the BCG antitumor response against murine melanoma. Our findings suggest that the mechanism involves the infiltration of antitumoral immune cells into the TME, promoting cytokine responses and cytotoxic activity due to MyD88-dependent cellular activation. Thus, our study contributes to a better understanding of the immune mechanisms involved in BCG immunotherapy in murine melanoma and support future approaches including the use of MyD88-related agonists associated with BCG to improve cancer immunotherapy.

## Data availability statement

The original contributions presented in the study are included in the article/**Supplementary Material**. Further inquiries can be directed to the corresponding author.

## Ethics statement

The animal study was approved by the Ethics Commission on Animal Use (CEUA) from UFMG under permit #372/2019. The study was conducted in accordance with the local legislation and institutional requirements.

## Author contributions

VB: Formal analysis, Investigation, Validation, Writing – original draft. FM: Data curation, Formal analysis, Investigation, Methodology, Writing – original draft. CC: Investigation, Methodology, Writing – original draft. ND: Conceptualization, Investigation, Methodology, Supervision, Validation, Writing – original draft, Writing – review & editing. SO: Conceptualization, Funding acquisition, Project administration, Supervision, Visualization, Writing – review & editing.

## Supplementary material

Supplementary Video 1 BCG intercellular localization inside B16-F10 tumor cells. B16-F10 were infected with BCG Pasteur expressing RFP (MOI 5). BCG (RFP) are shown in red, Phalloidin stained the cytoskeleton (green), and DAPI identify the nucleus (blue). The green color intensity was reduced and after increased in the video to represent the cytoskeleton involving bacteria. Confocal microscopy 3D images were captured by Nikon Eclipse Ti and rendered using Volocity 6.2 software.

Supplementary Video 2 BCG intercellular localization inside BMDMs cells. BMDMs were infected with BCG Pasteur expressing RFP (MOI 5). BCG (RFP) are shown in red, Phalloidin stained the cytoskeleton (green), and DAPI identify the nucleus (blue). The green color intensity was reduced and after increased in the video to represent the cytoskeleton involving bacteria. Confocal microscopy 3D images were captured by Nikon Eclipse Ti and rendered using Volocity 6.2 software.
