# Supplementary material for: Bacillus Calmette-Guérin immunotherapy induces an efficient antitumor response to control murine melanoma depending on MyD88 signaling
Source: Front Immunol. 2024 May 21;15:1380069. doi: 10.3389/fimmu.2024.1380069 (PMC11148268; doi:10.3389/fimmu.2024.1380069)
Supplement: Supplementary file 3 [file DataSheet_1.pdf]

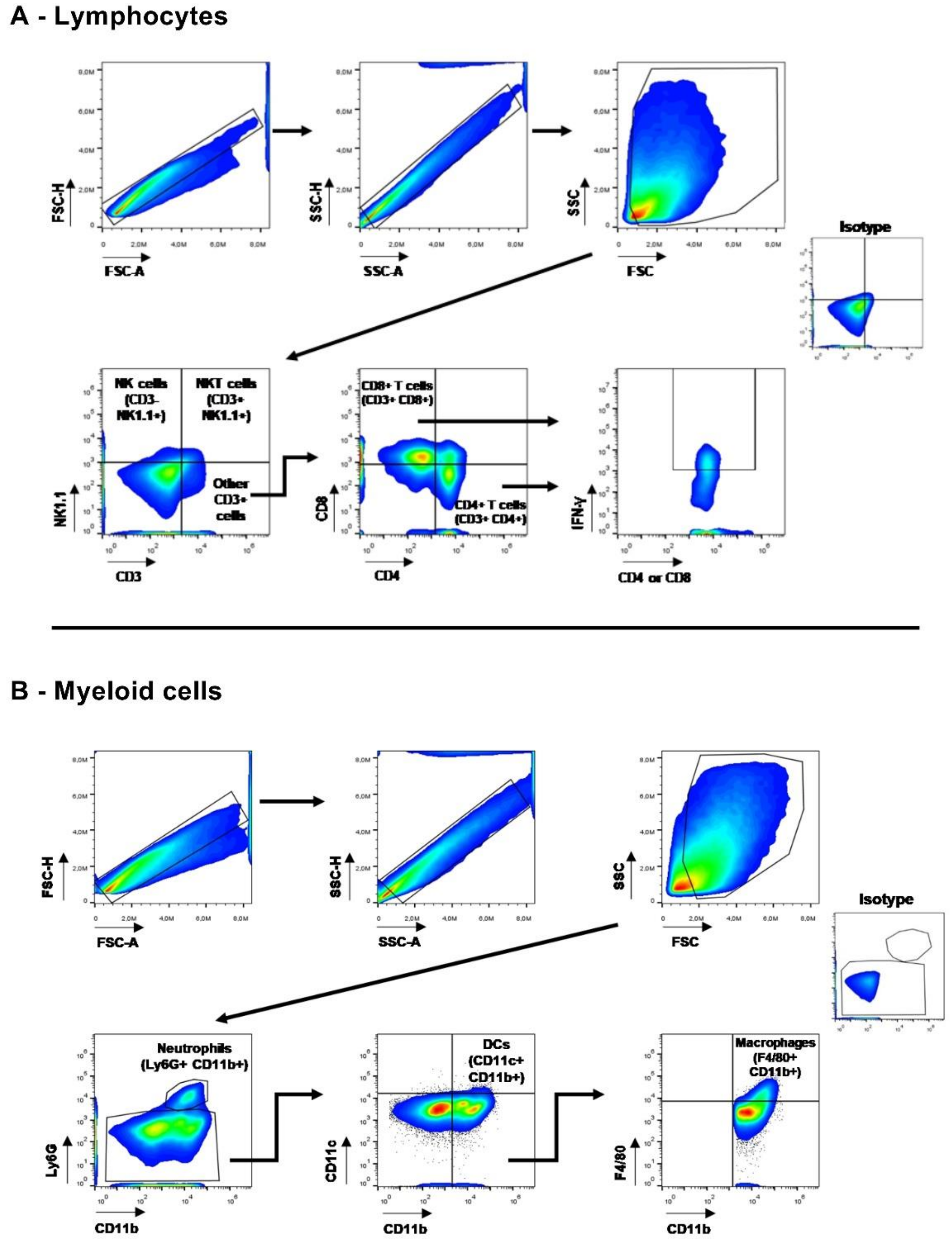

Supplementary Figure 1. Gating strategy for *ex vivo* flow cytometry evaluation of the inflammatory infiltrate associated with the TME from C57BL/6 and MyD88$^{-/-}$ tumors. **(A)** Lymphocytes panel: Total tumor cells population were gated for NK1.1^{+}CD3^{-} (NK cells) or NK1.1^{+}CD3^{+} cells (NKT cells). Following, the remaining CD3^{+} cells were gated as CD3^{+}CD4^{+} (CD4^{+} T cells) or CD3^{+}CD8^{+} (CD8^{+} T cells). IFN-γ expression was evaluated in CD4 and CD8 positive populations. **(B)** Myeloid cells panel: Total tumor cells population were gated for Ly6G^{+}CD11b^{+} (Neutrophils) and the reimaging cells were gated as CD11c^{+}CD11b^{+} (Dendritic cells). Following, the CD11c negative cells were gated for F4/80^{+}CD11b^{+} (Macrophages). The isotype controls are shown on the right side of the figures.

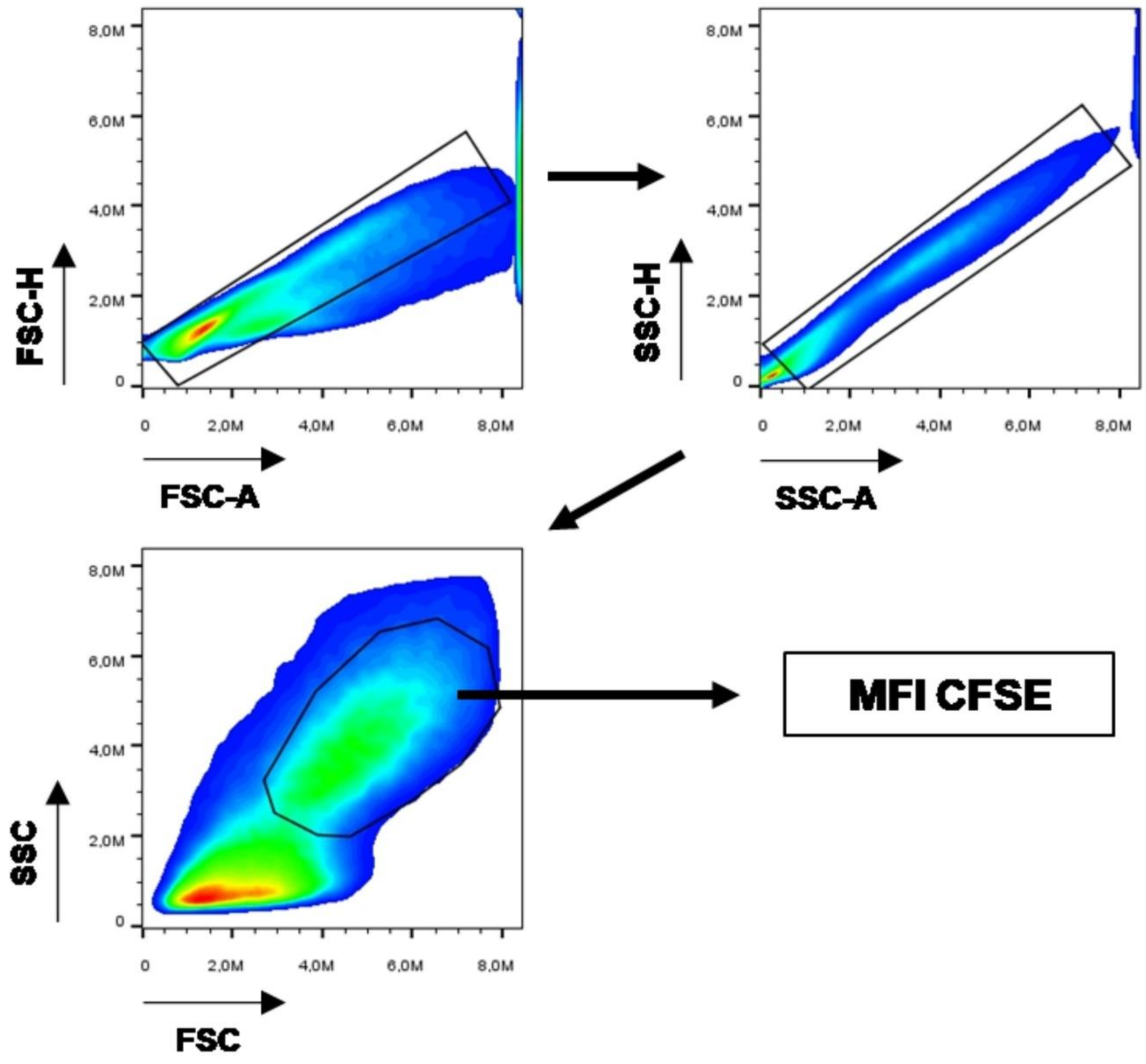

Supplementary Figure 2. Gating strategy for *in vitro* killing assay. The total cells from each well in the co-culture 24 well plates were transferred to 96 well plates and the CFSE stained B16-F10 cell population analyzed by the Mean Fluorescence Intensity (MFI) values of the CFSE staining.

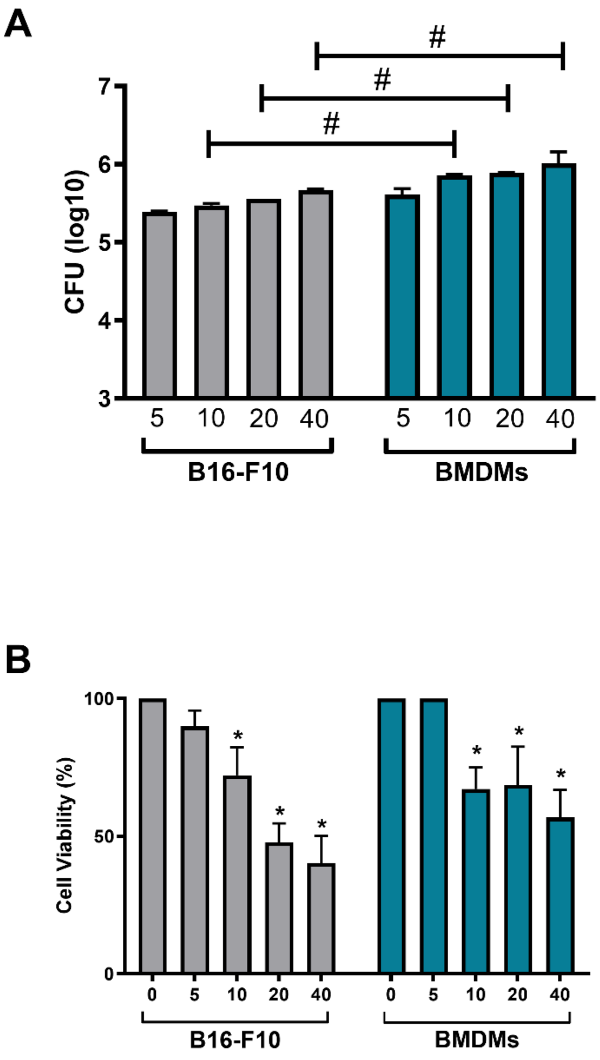

Supplementary Figure 3. BCG infectivity and cell viability. **(A)** B16-F10 cells and BMDMs were infected *in vitro* with BCG (MOI 5, 10, 20 and 40) for 24h. After this period, the cell cultures were washed twice with PBS to remove free bacteria and subsequently lysed, diluted and plated for quantification of the intracellular bacterial load by counting total CFU after 21-30 days. Bacterial loads (CFU) are represented in logarithmic scale. **(B)** Cell viability of B16-F10 tumor cells and BMDMs was evaluated using Trypan blue to distinguished live cells after 24 hrs of BCG infection (MOI 5, 10, 20, and 40). #Statistically significant comparing B16-F10 and BMDMs infected with the same MOI (ANOVA; $P \leq 0.05$). *Statistically significant compared to the respective not infected control (ANOVA; $P \leq 0.05$).
